# The Effects of Resveratrol-Rich Extracts of *Vitis vinifera* Pruning Waste on HeLa, MCF-7 and MRC-5 Cells: Apoptosis, Autophagia and Necrosis Interplay

**DOI:** 10.3390/pharmaceutics14102017

**Published:** 2022-09-23

**Authors:** Aleksandra Jovanović Galović, Nataša Jovanović Lješković, Senka Vidović, Jelena Vladić, Nikola Jojić, Milan Ilić, Tatjana Srdić Rajić, Vesna Kojić, Dimitar Jakimov

**Affiliations:** 1 Faculty of Pharmacy Novi Sad, University of Business Academy, Trg Mladenaca 5, 21000 Novi Sad, Serbia; 2 Faculty of Technology, Department of Biotechnology and Pharmaceutical Engineering, University of Novi Sad, Bulevar Cara Lazara 1, 21000 Novi Sad, Serbia; 3 Institute for Oncology and Radiology of Serbia, Department of Experimental Oncology, Pasterova 14, 11000 Belgrade, Serbia; 4 Oncology Institute of Vojvodina, Faculty of Medicine, University of Novi Sad, Put doktora Goldmana 4, 21204 Sremska Kamenica, Serbia

**Keywords:** resveratrol, autophagia, apoptosis, necrosis, cell culture, cancer prevention, subcritical water extract

## Abstract

Resveratrol is a well-studied plant-derived molecule in cancer biology, with a plethora of documented in vitro effects. However, its low bioavailability and toxicity risk hamper its wider use. In this study, vine shoots after pruning were used as a source of resveratrol (RSV). The activity of subcritical water extract (SWE) and dry extract (DE) is examined on three cell lines: HeLa, MCF-7 and MRC-5. The cytotoxic effect is assessed by the MTT test and EB/AO staining, levels of apoptosis are determined by Annexin V assay, autophagia by ULK-1 expression using Western blot and NF-kB activation by p65 ELISA. Our results show that both resveratrol-rich extracts (DE, SWE) have a preferential cytotoxic effect on malignant cell lines (HeLa, MCF-7), and low cytotoxicity on non-malignant cells in culture (MRC-5). Further experiments indicate that the investigated malignant cells undergo different cell death pathways. MCF-7 cells died preferentially by apoptosis, while the HeLa cells died most likely by necrosis (possibly ferroptosis). Protective autophagia is diminished upon treatment with DE in both HeLa and MCF-7 cells, while SWE does not influence the level of autophagia. The extracts are effective even at low concentrations (below IC_50_) in the activation of NF-kB (p65 translocation).

## 1. Introduction

Growing vines is one of the oldest and most important agricultural activities that occurs on over 7.6 million hectares in the world. The Mediterranean region and certain parts of continental Europe have a long tradition of viticulture dating back to ancient times. According to some estimates, for every 1.3 kg of wine that is produced during one year, about 2 × 10^7^ tons of pruning waste are collected. Pruning waste is one of the most important categories of waste in viticulture, and in recent years has been the subject of research aimed at finding innovative uses [1,2,3]. Young shoots of the vine that are periodically removed from the plant are characterized by a high content of cellulose (55%) and lignin (38.5%) [2]. However, it is the presence of phenolic and aromatic hydrocarbons with biological activity that may enable innovative uses of this organic waste [4,5]. The phenolic group of these compounds is known for its beneficial effects on human health [6,7]), including cancer prevention and treatment [8]. In this group, *trans*-resveratrol was identified as the compound with the most significant biological activity [9,10].

Resveratrol (RSV) is a stilbenoid polyphenol with a wide range of biological effects, antioxidant, cardioprotective, neuroprotective, anti-inflammatory and anticancer activities. This polyphenol is present in both *trans*- and *cis*-isomeric forms, trans being dominant in terms of its prevalence and therapeutic application. The anticancer effects of *trans*-resveratrol have been well documented. Numerous studies have demonstrated that it is able to inhibit all stages of carcinogenesis (e.g., initiation, promotion and progression) and induce cellular responses, such as cell cycle arrest, differentiation and apoptosis, and to enhance cancer cells’ anti-proliferation. Resveratrol showed proliferation-inhibitory (inducing cell shrinkage) and apoptosis-inducing effects in HeLa cells through the activation of caspases-3 and -9, upregulation of the expression of the pro-apoptotic B-cell lymphoma (Bcl)-2-associated X protein and downregulation of the expression of the anti-apoptotic proteins Bcl-2 and Bcl extra large, as well as increased expression of p53 [11]. The inhibitory effects of RSV on histone deacetylase led to cell cycle arrest, apoptosis and autophagy, angiogenesis inhibition, increasing reactive oxygen species generation, thus causing oxidative stress and mitotic cell death in cancer cells [12]. Resveratrol as a chemopreventive and chemotherapeutic agent suppresses angiogenesis, invasion and metastasis by inhibiting hypoxia-induced factor-1α (HIF-1α) and matrix metalloproteinases (MMPs). Resveratrol also targets hormone signaling due to its potent antiestrogenic activity in hormone-dependent cancers [13]. Furthermore, it has been shown that RSV may reverse drug resistance and reduce the risk of multidrug resistance (MDR) via multiple targets related to carcinogenesis and chemo/radioresistance [14].

Among its numerous effects [15], it has been shown that resveratrol inhibits the progression of cervical cancer by suppressing the expression of HPV E6 and E7 genes [16], decreases angiogenic activity by inhibiting the expression of HIF-1α and VEGF through blocking ERK1/2 and PI3K/Akt signaling pathways [17] and inhibits the migration of HeLa cells by suppression of NF-kB and AP-1-mediated MM9 expression [18]. The involvement of natural polyphenols, including resveratrol, in the chemoprevention of cervical cancer was recently reviewed [19,20]. Resveratrol is considered as one of the important nutraceuticals of the Mediterranean diet with its particular importance in breast cancer prevention and treatment [21].

It was demonstrated that subcritical water can be used successfully for the recovery of stilbenes from grapevine canes, woods and roots [22], and resveratrol from grape seeds [23]. The subcritical water extraction represents a new green extraction technology that uses water as a solvent at temperatures between the boiling point 100 °C and critical point 374.15 °C. For the water to remain liquid, it is necessary to apply pressure. Under these conditions, changes in the physico-chemical characteristics of water occur, such as the decrease in the dielectric constant, decrease in viscosity and increase in diffusivity. In adequate conditions, subcritical water possesses a dielectric constant similar to organic solvents. Therefore, by varying the extraction process conditions, it is possible to adjust the selectivity towards certain components and in that way achieve an efficient extraction of components of different polarities [24]. However, compared to the liquid extracts, dry extracts provide greater stability and can be incorporated further into final products, such as tablets and pills. In addition, dry extracts have economic advantages, such as a lower volume, which decreases transportation and storage costs [25]. The aim of this study is to evaluate the biological effect of pruning waste extracts, rich in *trans*-resveratrol, obtained by the subcritical water extraction process (subcritical water extract, SWE) and subsequently dried (dried extract, DE) on cervical and breast cancer cells in culture in comparison with pure *trans*-resveratrol. Levels of cytotoxicity, apoptosis, autophagy and NF-kB activation are determined.

## 2. Materials and Methods

### 2.1. Material

Pruning *Vitis vinifera* material was collected from the vineyards of Vojvodina, Serbia.

### 2.2. Subcritical Water Extraction 

Subcritical water extraction was performed in a 2 dm^3^ batch-type high-pressure extractor (Parr 4520, Moline, IL, USA). The reactor was equipped with an anchor stirrer and electric heater, which allowed the heating of the reaction mixture up to 350 °C. In all experimental runs, the material was mixed with water in a 1:10 (g/mL) ratio. Nitrogen was injected into the extractor to prevent possible oxidation at high temperatures in the presence of oxygen from the air. After the extraction, the extractor was immediately cooled to reach room temperature, and nitrogen was discharged from the extractor. The extracts were filtered through filter paper under vacuum, collected into glass flasks and stored at 4 C in a dark place until analysis.

The extractions were conducted at temperatures of 120–200 °C, extraction time of 15–35 min and the addition of HCl 0–1.5%, at a constant pressure of 30 bar. A Box–Behnken design with three numeric factors on three levels (Supplementary Materials Table S1) was used for the design of the experiments. The design consisted of fifteen randomized runs with three replicates at the central point. Independent variables were coded in a range from −1 to 1 (temperatures: 120 °C (−1), 160 °C (0), 200 °C (1); extraction times: 15 min (−1), 25 min (0), 35 min (1); HCl additions: 0% (−1), 0.75% (0), 1.5% (1). To determine the optimal conditions of the extraction and obtain the maximal content of total phenols, total flavonoids and antioxidant activity, the optimization was conducted using the Design Expert 13 Trial (Stat-Ease, Minneapolis, MN, USA). The liquid extract obtained under optimal extraction conditions was used in this study. 

### 2.3. Drying of the Liquid Extract

The pilot-scale spray dryer (APV Anhydro AS, Søborg, Denmark) was used for converting the optimal liquid extract of pruning waste into dry extract. The speed of the atomizer was 20,000–21,000 rpm. The inlet air temperature was 120 °C, while the outlet temperature was in the range of 75–80 °C. Maltodextrin (dextrose equivalent 16.5–19.5) was used as a carrier for drying the liquid extracts (50% related to the weight of total solids (*w*/*w*)). The feed mixture was fed into the spray drying system using a peristaltic pump (flow rate of 4.3 L/h). The dry extract was separated from the air stream using the cyclone separation method. After collection, it was stored in glass packaging, in a desiccator, until further analysis.

### 2.4. Determination of Total Phenol and Total Flavonoids Content

Total phenol content in the obtained extracts was measured according to the Folin–Ciocalteu procedure described by [26]. Absorbance was measured at 750 nm (6300 Spectrophotometer, Jenway, Staffordshire, UK). The calibration curve was defined using gallic acid as a standard and the results were expressed as mg of gallic acid equivalents (GAEs) per g of dry weight or dry extract. To determine the total flavonoid content, an aluminum chloride colorimetric assay was used [27]. The absorbance was measured at 510 nm. The total flavonoid content in extracts was expressed as mg of catechin equivalents (CEs) per g dry weight or dry extract through the standard calibration curve defined with catechin. All measurements were performed in triplicate.

### 2.5. Determination of Antioxidant Activity

The antioxidant activity of extracts was analyzed using the 2,2-diphenyl-1-picrylhydrazyl (DPPH) assay [28]. Different volumes of extracts were mixed with 95% methanol solution and 90 μM DPPH solution. After the 60 min incubation period at room temperature, absorption was measured at a wavelength of 515 nm. The antioxidant activity was expressed as an IC_50_ value, which represents the concentration of the extract that inhibits 50% DPPH radicals. All the measurements were performed in triplicate.

### 2.6. Resveratrol Content Determination—High-Performance Liquid Chromatography with Diode Array Detection (HPLC-DAD) Analysis 

Acetonitrile and acetic acid for HPLC analysis were of HPLC grade. Water was prepared by a Millipore Simplicity water purification system (Millipore, Bedford, MA, USA). The resveratrol standard was purchased from Sigma Aldrich (Steinheim, Germany). 

HPLC analysis was performed using an Agilent 1200 RR chromatograph with UV-Vis Diode Array Detector (Agilent Technologies, Santa Clara, CA, USA). The chromatographic separation was achieved on a Zorbax Sb-Aqua column (250 mm × 4.5 mm, 5 µg, Agilent Technologies, Santa Clara, CA, USA). A gradient consisting of solvents A (CH_3_COOH/H_2_O, 5:95, *v*/*v*) and B (acetonitrile) was applied at a flow rate of 0.5 mL/min as follows: 0 to 60% B linear from 0 to 35 min, and 60 to 100% B linear from 35 to 40 min. Chromatograms were acquired at 306 nm. The content of resveratrol was determined using calibration curves of authentic standards of resveratrol. The analysis was conducted in triplicate and the results were expressed as the mean value ± standard deviation.

### 2.7. Cell Lines

The cell lines used in the study were MCF-7 (ATCC HTB 22, human breast adenocarcinoma ER+), HeLa (ATCC CCL 2, cervix epithelioid carcinoma) and MRC-5 (ATCC CCL 171, normal fetal lung fibroblasts). The cells were grown in Dulbecco′s modified Eagle′s medium (DMEM) with 4.5% of glucose, supplemented with 10% of fetal calf serum (FTS, Sigma-Aldrich, St. Louis, MO, USA) and antibiotics and a antimycotics solution (Sigma-Aldrich, St. Louis, MO, USA). The cell line was cultured in flasks (Costar, 25 cm^2^) at 37 °C in an atmosphere of 100% humidity and 5% of CO_2_ (Heraeus, Hanau, Germany). Exponentially growing viable cells were used throughout the assays.

### 2.8. Treatment of Cells and IC_50_ Determination

The treatment of cells was conducted by extracts (DE and SWE), resveratrol and maltodextrin, since maltodextrin was used as the carrier in spray drying. Obtained *V. vinifera* pruning waste extracts were used in a serious of concentrations ranging from 25–125 μg/mL in order to define the IC_50_ concentration for the period of incubation of 48 h. Extract solubilization was performed in DMSO by making a 10x stock solution. The diluted extracts’ solutions were added in volumes of 10 μL/well to attain the designated concentrations.

Arzanol was purchased from Sigma (CAS No.32274-52-5; PubChem Substance ID: 329824643) and dissolved prior to treatment in DMSO, to create a stock solution. Subsequently, the stock solution was diluted to attain the desired concentration. The diluted arzanol solution was added in a volume of 10 μL per well.

### 2.9. MTT Assay

Growth inhibition was evaluated by tetrazolium colorimetric MTT assay (Sigma-Aldrich, St. Louis, MO, USA) [29,30]. The assay is based on the cleavage of tetrazolium salt MTT (3-(4,5-dimethylthiazol-2-yl)-2,5-diphenyl tetrazolium bromide) to formazan by mitochondrial dehydrogenases in viable cells. Exponentially growing cells were harvested, counted by trypan blue and plated into 96-well microtiter plates (Costar) at an optimal seeding density of 10 × 10^3^ cells per well to assure a logarithmic growth rate throughout the assay period. Viable cells were plated in a volume of 90 µL per well and pre-incubated in complete medium at 37 °C for 24 h to allow cell stabilization prior to the addition of substances. Tested substances, at tenfold the required final concentration, were added (10 µL/well) to all wells, except to the control ones, and microplates were incubated for 24 and 48 h. The wells containing cells without tested substances were used as controls. Three hours before the end of the incubation period, 10 µL of MTT solution was added to all wells. MTT was dissolved in the medium at 5 mg/mL and filtered to sterilize and remove a small amount of insoluble residue present in some batches of MTT. Acid-isopropanol (100 µL of 0.04 N HCl in isopropanol) was added to all wells and mixed thoroughly to dissolve the dark-blue crystals. After a few minutes at room temperature, to ensure that all crystals were dissolved, the plates were read on a spectrophotometer plate reader (Multiscan MCC340, Labsystems) at 540/690 nm. The wells without cells containing complete medium and MTT only acted as blanks.

The inhibition of growth was expressed as a percent of the control, and cytotoxicity was calculated according to the formula: (1 − A_test_/A_control_) × 100. The substance potency was expressed as IC_50_ (50% inhibitory concentration). All experiments were replicated three times and the results were expressed as mean values.

### 2.10. Double Fluorescent Staining

We performed the double-staining fluorescence method for testing the apoptosis induction potential of the investigated extracts and derivatives. The presence of cells in apoptosis was detected on the preparations by staining with ethidium bromide and acridine orange (EB/AO) fluorescent dyes. After 24 h of treatment with the IC_50_ concentrations of the tested compounds, the samples of HeLa and MCF-7 cells were stained with two fluorescent dyes, ethidium bromide and acridine orange, in order to detect cells with disturbed membrane integrities. The untreated cell cultures were used as control samples. Following staining, the specimens were examined with a fluorescence microscope (Olympus BX51). Microphotographs of fluorescent signals were created with a digital camera (Olympus CAMEDIA 3040) mounted on the microscope for each specimen. The images were analyzed in the *ImageJ* computer program (NIH Image, http://imagej.nih.gov (accessed on 5 March 2022)). We compared the samples by their red-to-green signal ratio, measuring the density of the separated red-and-green-image color channels.

### 2.11. Flow-Cytometric Analysis of Cell Cycle Phase Distribution

Briefly, 2 × 10^5^ cells/Petri dish (dimensions 60 × 15 mm, NUNC) were treated with investigated compounds as indicated. Following collection, the cells were fixed with ethanol. The fixed cells were washed with PBS and incubated with RNase A (1 mg/mL) for 30 min at 37 °C. Just prior to flow-cytometric analysis, the cells were stained with propidium iodide (PI) at a concentration of 400 μg/mL (Sigma-Aldrich, St. Louis, MO, USA). The cell cycle phase distribution was analyzed by an FACS Calibur Becton Dickinson flow cytometer using Cell Quest computer software (Becton Dickinson, Heidelberg, Germany).

### 2.12. Apoptotic Assay

Apoptotic rates were assessed with flow cytometry using the Annexin V fluorescein isothiocyanate/propidium iodide kit (BD Pharmingen, San Diego, CA, USA). Samples were prepared according to the manufacturer’s instructions. Flow cytometry analysis was performed using an FACS Calibur cytometer using Cell Quest computer software (Becton Dickinson, Heidelberg, Germany).

### 2.13. Quantification of Mitochondrial Transmembrane Potential

The mitochondrial transmembrane potential (Δψm) was measured using a cationic fluorochrome JC-1 (5′,6,6′-tetrahloro-1,1′,3,3′-tetraethylbenzimidazolylcarbocyanine iodide) (Sigma-Aldrich, St. Louis, MO, USA), as described by [31] (Flow Cytometric Analysis of Mitochondrial Membrane Potential Using JC-1. Curr. Protoc. Cytom, 13, 9.14.1–9.14.7). Briefly, 1 × 10^6^ cells resuspended in 160 μL of phosphate-buffered saline were stained with 40 μL of JC-1 (15.5 µM) for 15 min at 37 °C. After washing, the samples were analyzed by flow cytometry using Cell Quest software (Becton Dickinson, Heidelberg, Germany).

### 2.14. Western Blotting

The protein concentration in cell lysate was determined by Bradford protein assay (Bradford, M. M. 1976) in a 96-well microtiter plate (ThermoLab Systems, Multiscan Accent spectrophotometer) using bovine serum albumin as the standard. Molecular mass markers for proteins were obtained from Amersham Biosciences (Amersham, UK).

For the Western blotting, 50 μg of proteins per sample were separated by electrophoresis and electrotransferred to a polyvinylidene difluoride (PVDF) membrane Hybond-P (Amersham Biosciences, Arlington Heights, IL, USA) and then blotted with primary antibodies (ULK1 and Actin). Monoclonal antibodies against human ULK1 were obtained from Thermo Fisher Scientific (Waltham, MA, USA). Protein b-actin was used as an experimental internal control (Sigma-Aldrich, St. Louis, MO, USA). Blots were developed with an enhanced chemiluminescence (ECL Plus) detection kit (Amersham Biosciences), including peroxidase-labeled secondary antibodies for the detection of proteins. Chemiluminescent signals were recorded on Hyperfilms (Amersham Biosciences) and photographed with a digital camera. Images of protein expression were analyzed following minor level adjustments in the *ImageJ* computer program (NIH Image, http://imagej.nih.gov (accessed on 5 March 2022)). The expression of apoptotic proteins was analyzed by densitometry and compared to the control sample.

### 2.15. NFkB p65 Transcription Factor ELISA

Nuclear extracts of all three cell lines (HeLa, MRC-5, MCF-7) were obtained with a Nuclear Extraction Kit (ab113474, Abcam, Cambridge, UK), according to the manufacturer’s instructions. Protein concentration was determined prior to the ELISA NF-kB p65 assay, using BCA a Protein Quantification Kit (Abcam, ab102536), while the NFkB p65 transcription factor was analyzed with an ELISA kit (Abcam, ab133112).

### 2.16. Statistical Analysis

Two-way ANOVA was performed on type of extract versus concentration for each cell line and incubation time (24 h and 48 h). Additionally, two-way ANOVA was performed on type of extract versus cell type for each concentration (25 M–125 M) (Supplementary Materials Tables S2–S18).

## 3. Results

### 3.1. Content of Total Phenols, Total Flavonoids and Antioxidant Activity of Extracts

To provide an effective valorization of the vine pruning waste, subcritical water extraction was used. The extracts were obtained under different conditions of extraction, temperature (120–200 °C), extraction time (15–35 min) and the addition of HCl (0–1.5%), at a constant pressure of 30 bar. The obtained extracts were examined in relation to the content of total phenols, total flavonoids, as well as antioxidant activity (Supplementary Materials Table S1). Based on the obtained results, a numerical optimization of the subcritical water extraction was conducted to attain a liquid extract with the highest content of total phenols, total flavonoids and antioxidant activity. It was determined that the optimal conditions of the extraction were: temperature: 200 °C, extraction time: 18.27 min and HCl addition: 1.5%. By applying these conditions, a liquid extract was obtained with the content of total phenols of 10.667 (g CE/100 g DW), total flavonoids: 2.909 (g CE/100 g DW) and IC_50_ value: 0.047 mg/mL. The optimal liquid extract was dried further using the spray drying method with the addition of maltodextrin. Moreover, the dry extract’s content of total phenols 75.56 GAE mg/100 g extract, total flavonoids 18.42 mg CE/g extract and IC_50_ value 0.121 mg/mL were determined (units are defined in the Materials and Methods Section 2.4: “Determination of total phenol and total flavonoids content”).

### 3.2. HPLC-DAD Analysis

The content of the resveratrol determined by HPLC-DAD analysis in the subcritical water extract (SWE) was 296.98 μg/100 mL of extract. While in the dry extract with maltodextrin (DE), the content of resveratrol was 57.19 μg/g of extract. In addition to resveratrol, traces of rutin and quercetin were identified in both types of extract.

### 3.3. MTT Test—Cytotoxicity on Normal and Malignant Cell Lines

HeLa, MCF-7 and MRC-5 cell lines were treated with DE (1), SWE (2), RSV (3) and maltodextrin (4) for 24 and 48 h in the concentration range of 20 to 125 µM. The cytotoxic effect on cells was measured by the tetrazolium colorimetric test (MTT test), immediately following the end of incubation with the test substance. Resveratrol (3) was the reference substance; maltodextrin (4) was a substance added during the preparation of dry extracts and had a negative control function.

HeLa cells are equally sensitive to all test compounds after 48 h. The reference resveratrol was more active than both test compounds against all cell lines at both test times. Maltodextrin (4) did not affect cell growth and did not present a cytotoxic effect in any cell line or in any of the examined time intervals.

None of the treatments presented significant effects on MRC-5, a healthy cell line, but they did present dose and time dependence on all the tested cell lines.

**Figure 1:**
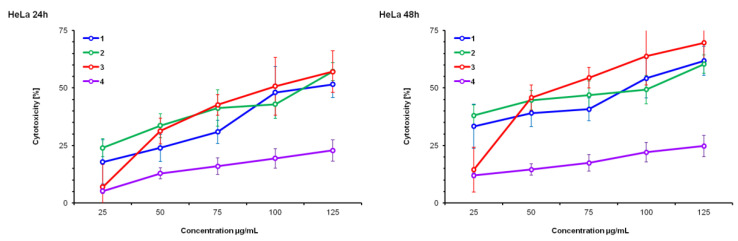
Cytotoxicity percentage determined by MTT test on HeLa, after 24 h and 48 h exposures to *V. vinifera* extracts in a concentration ranging from 25 μg/mL to 125 μg/mL. (1) Dry extract—DE; (2) subcritical water extract—SWE; (3) resveratrol—R; (4) maltodextrin—M.

**Figure 2:**
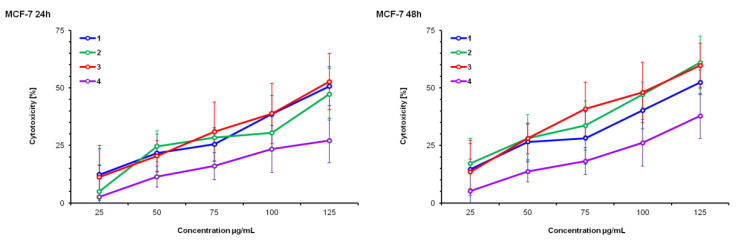
Cytotoxicity percentage determined by MTT test on MCF-7, after 24 h and 48 h exposures to *V. vinifera* extracts in a concentration ranging from 25 μg/mL to 125 μg/mL. (1) Dry extract—DE; (2) subcritical water extract—SWE; (3) resveratrol—R; (4) maltodextrin—M.

**Figure 3:**
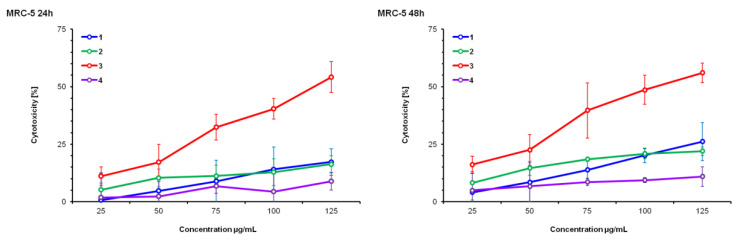
Cytotoxicity percentage determined by MTT test on MRC-5, after 24 h and 48 h exposures to *V. vinifera* extracts in a concentration ranging from 25 μg/mL to 125 μg/mL. (1) Dry extract—DE; (2) subcritical water extract—SWE; (3) resveratrol—RSV; (4) maltodextrin—M.

Based on the results presented in Figure 1, Figure 2 and Figure 3, as well as the IC_50_ values (Table 1), it can be observed that the HeLa cell line is the most sensitive to the action of the tested substances, followed by MCF-7, while MRC-5 cells are the least sensitive. This information is very important because MRC-5 cells are untransformed and are actually healthy connective tissue cells. The liquid extract obtained by subcritical water (2) presented greater activity against HeLa cells at both times than the dry extract (1). This difference can be explained by the weakening of biological potency due to the drying treatment, which involves high temperature, and it is therefore possible that a certain percentage of the active components of the extract were altered and therefore less biologically active. This trend was repeated in the inhibition of the growth of MCF-7 cells in the period of 48 h.

### 3.4. Double Fluorescent Staining

**Figure 4:**
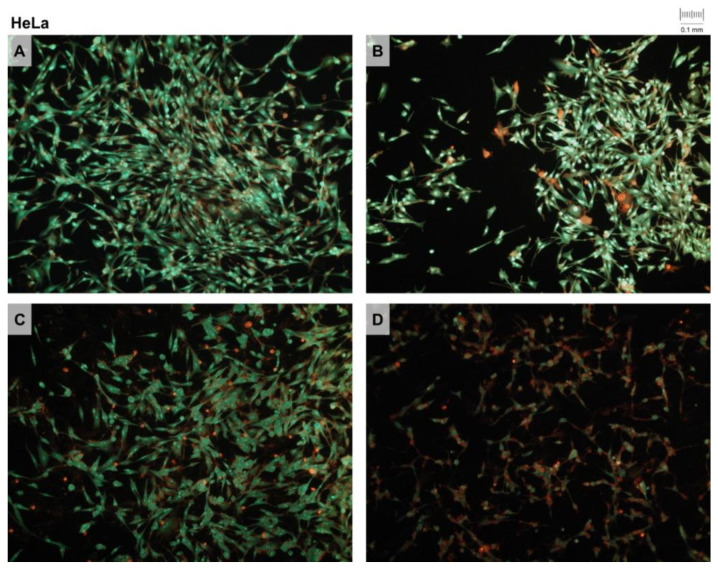
Double fluorescent staining of HeLa cell samples after 24 h treatment with extracts (DE, SWE) and resveratrol (RSV): control (**A**), dry extract (**B**), subcritical water extract (**C**) and resveratrol (**D**). Stained cells were photographed with an Olympus Camedia 3040 digital camera mounted on an Olympus BX51 microscope with a 10× magnification lens. Different intensities of fluorescent signals of the two colors can be observed.

**Figure 5:**
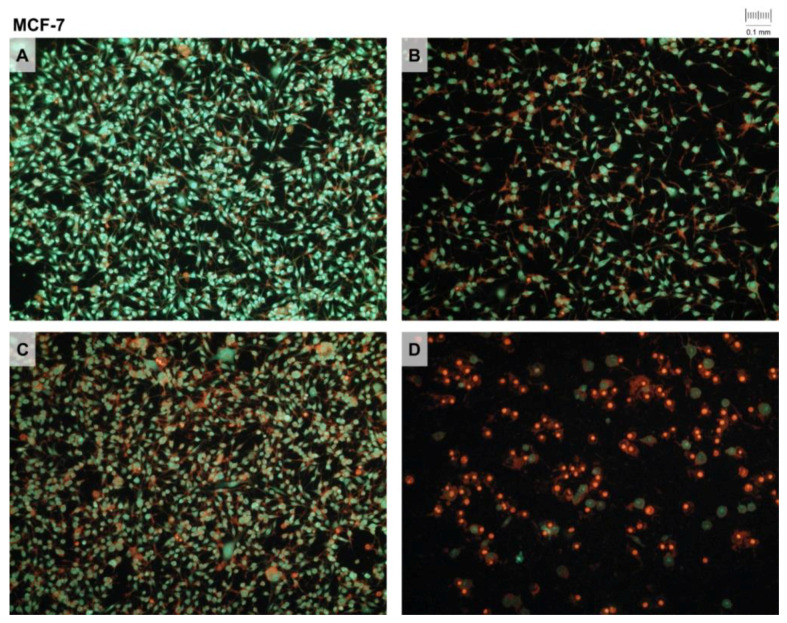
Double fluorescent staining of MCF-7 cell samples after 24 h treatment with extracts (DE, SWE) and resveratrol (RSV): control (**A**), dry extract (**B**), subcritical water extract (**C**) and resveratrol (**D**). Stained cells were photographed with an Olympus Camedia 3040 digital camera mounted on an Olympus BX51 microscope with a 10× magnification lens. Different intensities of fluorescent signals of the two colors can be observed.

The presence of membrane changes in cells that can be attributed to the apoptotic process induced by the action of the tested extracts was investigated on two cell lines, HeLa and MCF-7, using the method of double fluorescent staining (ethidium bromide and acridine orange). HeLa and MCF-7 cell samples were stained with two fluorescents after 24 h following treatment with IC_50_ concentrations of tested *V. vinifera* pruning waste extracts. Photomicrographs of the stained specimens of the control sample (**Ctrl**), DE (**1**), SWE (**2**) and RSV (**3**) are presented in Figure 4 and Figure 5.

**Figure 6:**
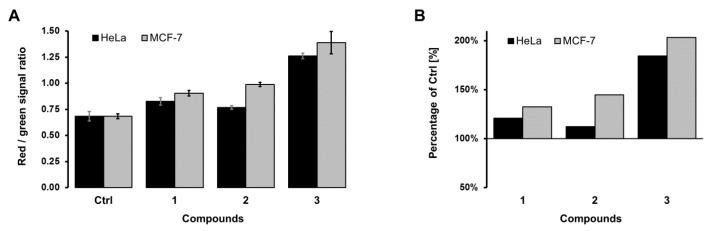
(**A**)—Densitometry results obtained by measuring the ratio of red and green signals of fluorescent colors on microphotographs of samples in the *ImageJ* computer program (NIH Image, http://imagej.nih.gov (accessed on 5 March 2022)). (**B**)—The results of the sample measurement, presented in relation to the control. Ctrl—control; 1—dry extract; 2—supercritical water extract; 3—resveratrol.

The results of the double fluorescence test, presented as the ratio of red and green fluorescent signal densities (measured using the *ImageJ* computer program), as well as their relation to the untreated control samples, are presented in Figure 6.

The results show that DE (**1**) and SWE (**2**) after 24 h of treatment induce approximately similar levels of apoptosis/necrosis, while RSV (**3**) produces a higher level of cell death. As for the cell type, it is evident that MCF-7 cells have overall higher levels of cell death upon 48 h of treatment with IC_50_ concentrations of extracts and RSV.

### 3.5. Flow Cytometry

**Figure 7:**
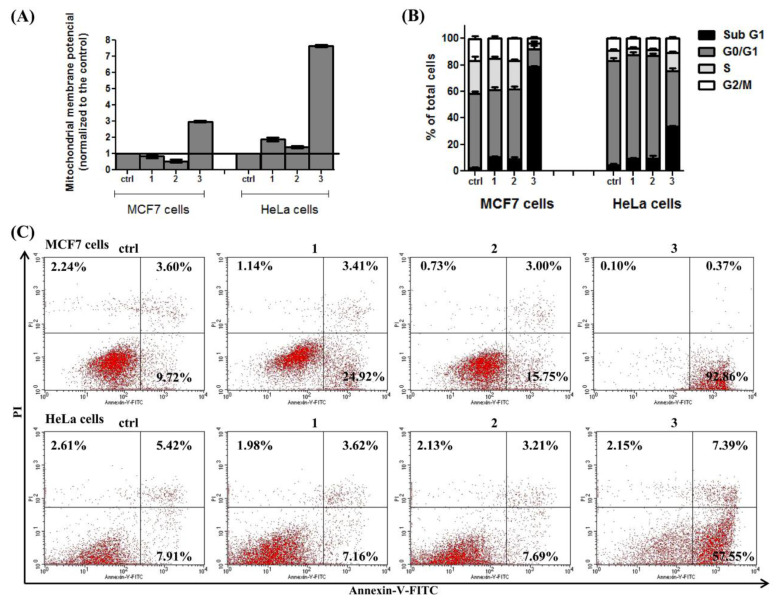
Flow cytometry analysis of MCF-7 and HeLa cells after 48 h of treatment with IC_50_ concentrations of extracts and resveratrol: (**A**) mitochondrial membrane potential, normalized to the control; (**B**) cell cycle phase distribution expressed as % of total cell number; (**C**) apoptosis rates assessed by the Annexin V fluorescein isothiocyanate/propidium iodide kit. Ctrl—control; 1—dry extract; 2—subcritical water extract; 3—resveratrol.

To asses this hypothesis in our extracts (DE, SWE) and RSV-treated cells, mitochondrial membrane potential (ΔΨm), cell cycle phase distribution and apoptosis were measured. As presented in Figure 7A, samples 1 (dry extract, DE) and 2 (subcritical water extract, SWE) have no significant effect on mitochondrial membrane potential, while sample 3 (RSV) significantly increases ΔΨ in both cell lines. This increased ΔΨm may be due to the higher amount and activity of respiratory chain complexes. The analysis of the cell cycle after 48 h of cell treatment with extracts and resveratrol (1—DE, 2—SWE; 3—RSV) of MCF-7 (ER positive) and HeLa (ER negative) cells showed an increased subGo peak in MCF-7 cells, suggesting a higher rate of apoptosis (Figure 7B). Apoptosis activation was confirmed by increased levels of Annexin V in ER-positive MCF-7 cells treated with tested compounds (Figure 7C). On the other hand, only RSV could induce apoptosis in HeLa cells, but to a lesser extent than in MCF-7 cells (Figure 7C).

### 3.6. Western Blot Analysis of ULK-1 Protein Expression

**Figure 8:**
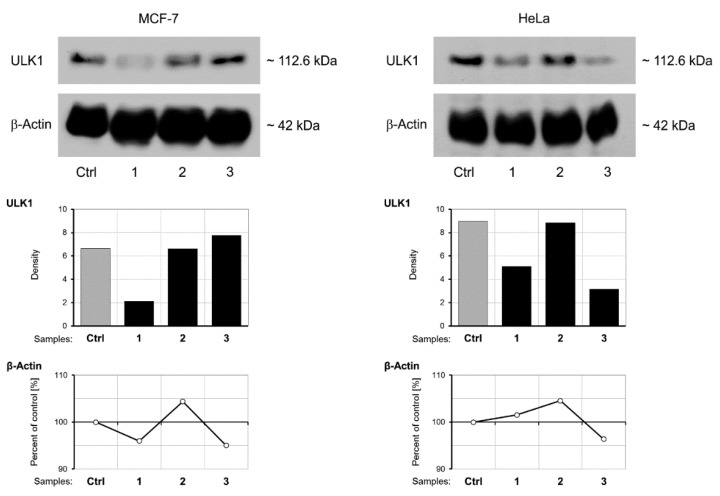
Western Blotting analysis of ULK-1 expression as a marker of autophagy in MCF-7 and HeLa cells after 48 h of treatment with IC_50_ concentrations of DE and SWE extracts and resveratrol. Ctrl—control; 1—dry extract; 2—subcritical water extract; 3—resveratrol.

Western blot analysis of ULK-1 protein expression in MCF-7 and HeLa cell samples was performed upon treatment with IC_50_ concentrations of extracts (DE, SWE) and RSV for period of 48 h. ULK-1 was chosen as a marker of autophagy. Of note was the relatively high expression in the control samples (Figure 8), compared to which the DE treatment produced a lower level of ULK-1 in both investigated cell lines. In HeLa cells, the same effect was produced by RSV treatment. SWE did not have an effect on ULK-1 expression.

### 3.7. NFkB p65 Transcription Factor ELISA

**Figure 9:**
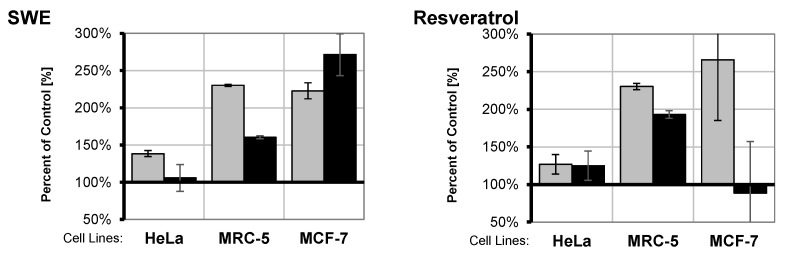
Activation of transcription factor NF-kB in the cells in the culture upon treatment with *V. vinifera* pruning waste subcritical water extract (SWE) and resveratrol in the concentration of 25 μg/mL (light gray), or 50 μg/mL (black) for 48 h. Results expressed as percent of control NF-kB p65 presence in nuclear extracts of HeLa, MRC-5 and MCF-7 cell lines, determined by ELISA p65 assay (Abcam, Cat. No. ab133112). Nuclear extracts obtained by Nuclear Extraction Kit (Abcam, Cat.No. ab113474). Protein concentration determined prior to ELISA NF-kB p65 assay using a BCA Protein Quantification Kit (Abcam, Cat. No. ab102536).

NF-kB p65 nuclear translocation was determined by ELISA in order to assess the changes in the activation of this transcription factor in HeLa, MRC-5 and MCF-7 cell lines upon treatment with two concentrations of SWE and RSV (Figure 9). The chosen concentrations were much lower than IC_50_ (see MTT test results). The results are presented as % of control. Under the investigated conditions, SWE produced the activation of NF-kB transcription factor in all three cell lines, which was less pronounced at higher concentrations in HeLa and MRC-5, while more pronounced in MCF-7. SWE and RSV had similar effects on p65 nuclear translocations in HeLa and MRC-5 cell lines, while MCF-7 responded differently. RSV did not induce NF-kB activation at higher concentrations (50 μg/mL) in MCF-7 cells.

## 4. Discussion

Breast cancer is the most common malignancy and the leading cause of cancer-linked deaths in women worldwide. According to the WHO data, there were 2.3 million women diagnosed with breast cancer and 685.000 deaths in 2020 globally [32]. The estimated age-standardized incidence rate is 47.8 per 100,000 [33], which is the highest of all cancer types. There are several types of breast cancer, luminal-like (luminal A and luminal B subtypes), characterized by the expression of estrogen (ER) and progesterone (PR) receptors, and basal-like breast cancer, characterized by the absence of ER, PR and human epidermal growth factor receptor 2 (HER2). The last one is often designated as triple-negative breast cancer (TNBC), difficult to treat due to the absence of molecular targets; hence, having the highest recurrence rate and lowest overall survival rate.

Cervical cancer is the fourth most common cancer in women, with an estimated 604,000 new cases and 342,000 deaths in 2020 globally. About 90% of the new cases and deaths worldwide in 2020 occurred in low- and middle-income countries [34]. Conventional cancer treatments are frequently hampered by drug resistance, which leads to recurrence and metastasis. In addition to the development of new drugs with a higher efficacy and less side effects, it seems important to increase the use of plant-derived products that have been proven to reduce resistance and sensitize the tumor cells, often modifying the tumor microenvironment (TME) and reprogramming cancer stem cells [35]. To that end, several authors reported findings on resveratrol effects as a supplement drug/substance to chemotherapeutic anticancer drugs: temozolomide (TMZ) [36]; doxorubicin (Doxo) [37,38]; cisplatin [39]; thymoquinone [40] and Adriamycin [41,42]. In these studies, RSV enhanced the sensitivity of the cancer cells, preventing chemoresistance. Moreover, these findings reveal molecular mechanisms that could be used for novel combined cancer therapies, which include resveratrol, especially for breast cancer.

Resveratrol (3.5.40-trihydroxystilbene) is one of the most-studied plant-derived molecules in cancer biology and was recently reviewed [15,21,43]. Although it is proven that RSV possesses many in vitro beneficial effects, in vivo effects must be considered in the perspective of its low bioavailability. After oral consumption, 77–80% is absorbed in the intestine mainly by passive diffusion or by forming complexes with membrane transporters [44]. Due to the fact that resveratrol has rapid and extensive metabolism, only trace amounts of unmodified resveratrol are detected in systemic circulation. The biotransformation of resveratrol occurs in the liver via phase II metabolism, leading to the formation of biologically active resveratrol metabolites. Upon liver metabolism and release into the bloodstream, resveratrol can be found mainly as a glucuronide and sulfate, and only trace concentrations of free RSV can be found in systemic circulation. Resveratrol undergoes rapid excretion, mainly via urine [45].

In the light of its low bioavailability combined with the high potential as an anticancer preventive or supplement drug, RSV-rich extracts are worth considering. In terms of prevention and long-term supplementation for the general population, plant extracts rich in bioactive compounds may be one of the approaches. At their core, the plant extracts mimic food, which could imply lower toxicity and improved bioavailability. Polypharmacology is a very complex discipline; however, it may mean a lower possibility for the disruption of a delicate balance in intricate molecular signaling pathways, the pathways that lie at the crossroads of a healthy and malignant cell. 

### 4.1. MTT Test

The MTT test results obtained in our experiments show that both extracts (DE, SWE) have cytotoxicity levels similar to those produced by resveratrol in malignant cell lines (HeLa and MCF-7), while significantly lower than non-malignant cells (MCR-5). This finding seems important in the context of supplementation, especially given the well-established toxicity of pure resveratrol at higher doses. In addition, IC_50_ data show that extracts are the most toxic to HeLa cells (lowest IC_50_), and that this effect is time-dependent in all cell lines. Based on the IC_50_ values, SWE was more potent than DE (the lowest IC_50_ after 48 h of treatment), which was expected since SWE contained a higher concentration of resveratrol, proven to be a potent anti-cancerous agent [6,7].

### 4.2. Double Fluorescent Staining

Fluorescent staining with ethidium bromide and acridine orange (EB/AO) showed a slightly higher apoptosis/necrosis levels in HeLa cells treated with DE, while for MCF-7 cells, SWE was more effective. These results are slightly different from the MTT test, since they show a higher percentage of apoptosis/necrosis in MCF-7 cells for all treatments. EB/AO staining was performed after 24 h of treatment of IC_50_ concentrations of DE, SWE and RSV, and presented cells with disturbed membrane integrities. The differences between the two methods accounted for this discrepancy, and showed that MCF-7 cells had undergone cell death that involved changes in the membrane integrity. This is plausible given the results of flow cytometry, which detected apoptosis in MCF-7 cells after treatment.

### 4.3. Flow Cytometry

The treatment of malignant cells (MCF-7 and HeLa) with IC_50_ concentrations of DE, SWE and RSV in the period of 48 h produced effects on the mitochondrial membrane potential (ΔΨm), cell cycle distribution and apoptosis (Figure 7). As was expected, RSV had a much more prominent effect an all parameters investigated by flow cytometry. However, SWE had a significant effect on MCF-7 cells. It lowered the mitochondrial membrane potential and induced apoptosis compared to the control samples. HeLa cells reacted in a different manner: both DE and SWE produced a higher mitochondrial membrane potential compared to MCF-7 cells, while RSV increased severalfold. The extracts did not induce apoptosis in this cell line. Apoptosis in HeLa cells was detected only upon treatment with RSV, based on the results obtained. It seems that HeLa cells underwent a different cell death program, given the rise in ΔΨm, possibly ferroptosis [46,47].

It is important to note that RSV is a phytoestrogen compound able to bind to estrogen receptors (ERs), and that MCF-7 cells are ER positive, while HeLa are ER negative. In that context, it seems that ERs are one of the targets for DE- and SWE-induced cell death in MCF-7 cells (increased subGo peak and increased levels of Annexin V). Additionally, it cannot be overlooked that ER is one of resveratrol’s targets.

### 4.4. Western Blot Analyses of ULK-1

It is known that serine/threonine protein kinases—AMP-activated protein kinase, mammalian target of rapamycin (mTOR) and unc-51-like kinase 1/2 (Ulk1/2)—are three closely connected junctions in the signaling network that regulates autophagy [48]. The perspectives and influences of natural compounds on autophagy in cancer were renewed [49]. The effect of our extracts (DE, SWE) on the level of autophagy in malignant cell lines (breast and cervical) was assessed by the Western blot analysis of the ULK1 protein. It was proven that the inhibition of mTOR activity and presence of ULK1 was crucial in resveratrol-induced autophagy in MCF-7 cells [50]. In our experiments, however, the basal expression (Ctrl) of ULK1 was considerably high in both HeLa and MCF-7 cells, which could imply a relatively high level of autophagy in these cells continuously grown in the culture (Figure 8). Taking into account the fact that autophagy may enable better cell survival and may, under certain conditions, be considered as an antagonist of apoptosis [48], this finding seems to be logical. In gliomas, resveratrol suppressed cytoprotective autophagy [36]. Thus, the decrease in ULK1 upon treatment with DE in both cell lines seems important to note. Interestingly, this decrease was even more pronounced in RSV-treated HeLa cells, in general less responsive in our set of experiments. This again suggested that signaling pathways triggered by our extracts and RSV were quite different in HeLa and MCF-7 cells. The interplay between autophagy and apoptosis is very complex and cell specific, meaning that the mechanisms of cell death in HeLa cells upon treatment with RSV and DE may involve lower levels of autophagy. SWE appeared to have no impact on autophagy, based on the Western blot results.

### 4.5. NFkB p65 Transcription Factor ELISA

In terms of the anti-inflammatory effects of resveratrol, this naturally occurring polyphenol demonstrated the ability to regulate pro- and inflammatory cytokines and chemokines, mainly by upregulating SIRT1, suppressing NF-κB and the associated cascades as well as inhibiting NALP3 inflammasome activation [51].

In order to assess whether a resveratrol-rich extract enters the nucleus and has any effect on the NF-kB in MCF-7, HeLa and MRC-5 cells, low concentrations (below IC_50_) of SWE extract were used (Figure 9). SWE was chosen for this experiments since it contained more resveratrol compared to the DE (Section 3.2. HPLC-DAD Analysis) and because its liquid state enabled more accurate dilutions. For comparison, the same concentrations of RSV were applied. Both concentrations (25 μg/mL and 50 μg/mL of SWE) produced the activation of NF-kB transcription factor in the investigated cell lines, although higher concentrations of the extracts produced a lower activation in HeLa and MRC-5 cells. A similar pattern was detected upon treatment with RSV. Changes in the NF-kB activation were most pronounced in MCF-7 cells, being concentration-dependent when cells were treated with SWE, while RSV produced activation at 25 μg/mL, which did not occur at 50 μg/mL (p65 level at approximately control level). The obtained results suggest the possible bi-phase effect of extracts and RSV under our experimental conditions—activation at low concentrations and inhibition at higher concentrations.

Since NF-kB (nuclear factor kappa light chain enhancer of activated B cells) is a transcription factor involved in the regulation of more than 500 genes connected with various processes—inflammation, malignant transformation, cell death, proliferation, invasion and metastasis [35]—it is quite plausible that the level of activation/inhibition of NF-kB responded differently, not only to various signals and molecules, but also in opposite directions to the same molecule depending on the concentration. This transcription factor was located at the crossroads of many signaling pathways and was a subject of interest in many research fields, from the prevention to therapy of many malignant diseases [21,52,53,54,55].

## 5. Conclusions

Taken together, the results suggest that both of our extracts (DE, SWE) have preferential cytotoxic effects on malignant cell lines (HeLa, MCF-7) and low cytotoxicity on non-malignant cells in culture (MRC-5), all of which are more pronounced after prolonged treatment. Furthermore, in our study HeLa and MCF-7 cells underwent different cell death pathways. MCF-7 cells died preferentially by apoptosis, while HeLa cells probably died by necrosis (possibly ferroptosis). Protective autophagia was diminished upon treatment with DE in both HeLa and MCF-7 cells, while SWE did not influence the level of autophagia. Extracts were effective even at low concentrations (below IC_50_) in the activation of NF-kB (p65 translocation).

It was evident that a complex interplay of various cell death pathways, apoptosis, necrosis (ferroptosis) and autophagia was triggered upon resveratrol-rich extract treatments. In addition, we must note that our extracts contained, in addition to RSV, a mixture of other bioactive compounds (flavonoid and non-flavonoid polyphenols), and in that respect mimic food. With that in mind, it was certain that the investigated extracts had a poly-pharmacological effect, albeit with selective cytotoxicity towards malignant cells. We argued that indeed these poly-pharmacology effects may be the way of surpassing the easily attained toxicity levels of resveratrol, and even improve bioavailability. In any case, these findings offer a basis for further research on resveratrol-rich vine pruning extracts as a potential source for supplements and the prevention of malignant diseases.

## Figures and Tables

**Table 1:** IC_50_ values obtained by MTT test, after 24 h/48 h treatments of HeLa, MCF-7 and MRC-5 cells with dry extract, subcritical water extract, resveratrol and maltodextrin.

MTT Test	HeLa		MCF-7		MRC-5	
Incubation time	**24 h**	**48 h**	**24 h**	**48 h**	**24 h**	**48 h**
Dry extract (DE)	133.03	83.66	162.51	148.91	3446.06	936.12
Subcritical water extract (SWE)	113.79	78.18	178.73	102.47	3454.52	2903.50
Resveratrol (RSV)	96.67	65.85	143.14	100.16	135.01	109.76
Maltodextrin (M)	>1000	>1000	>1000	>1000	>1000	>1000

## Data Availability

Not applicable.

## Supplementary Materials

The following supporting information can be downloaded at: https://www.mdpi.com/article/10.3390/pharmaceutics14102017/s1, Table S1: Content of total phenols, total flavonoids and antioxidant activities (expressed as IC_50_ value) of extracts obtained by subcritical water extraction; Table S2: TWO-WAY ANOVA sample legend; Tables S3–S18: TWO-WAY ANOVA analyses.
