# Supplementary material for: The Effects of Resveratrol-Rich Extracts of Vitis vinifera Pruning Waste on HeLa, MCF-7 and MRC-5 Cells: Apoptosis, Autophagia and Necrosis Interplay
Source: Pharmaceutics. 2022 Sep 23;14(10):2017. doi: 10.3390/pharmaceutics14102017 (PMC9607132; doi:10.3390/pharmaceutics14102017)
Supplement: Supplementary file 1 [file pharmaceutics-14-02017-s001.zip › pharmaceutics-1860382-supplementary.pdf]

Supplementary data

Table S1. Content of total phenols, total flavonoids, and antioxidant activity (expressed as IC_{50} value) of extracts obtained by subcritical water extraction

Temperature (°C)	Extraction time (min)	HCl addition (%)	Total phenols (g GAE/100 g DW)[1]	Total flavonoids (g CE/100 g DW)[2]	IC_{50} (mg/mL)
160	15	1.5	7.52	1.89	0.0650
120	25	0	2.44	1.08	0.1010
120	15	0.75	4.42	1.60	0.0826
160	25	0.75	7.48	1.82	0.0667
160	25	0.75	7.77	1.97	0.0656
200	15	0.75	10.17	2.75	0.0502
200	35	0.75	9.77	2.90	0.0456
160	25	0.75	7.14	1.76	0.0723
200	25	0	8.07	2.57	0.0435
200	25	1.5	9.90	2.90	0.0451
160	35	0	4.28	1.32	0.0718
160	15	0	5.08	1.61	0.0605
120	25	1.5	3.94	1.22	0.0833
160	35	1.5	7.41	1.81	0.0722
120	35	0.75	3.93	1.22	0.0906

1 - mg of gallic acid equivalents per g dry weight, 2 - mg of catechin equivalents per g dry weight

Table S2. TWO WAY ANOVA sample legend

Sample 1	DE (dry extract with maltodextrin)
Sample 2	SWE (supercritical water extract)
Sample 3	Resveratrol
Sample 4	Maltodextrin (control)

Table S3. TWO WAY ANOVA analysis for 24h treatment: type of extract vs concentrations in HeLa Cells. Sample: (samples 1-4); columns: concentrations (25 -125uM)

ANOVA

Source of Variation	*SS*	*df*	*MS*	*F*	*P-value*	*F crit*
Sample	3805.901	3	1268.634	273.9923	2.09E-16	3.098391
Columns	5476.676	4	1369.169	295.7054	1.75E-17	2.866081
Interaction	958.767	12	79.89725	17.25576	5.56E-08	2.277581
Within	92.6036	20	4.63018			
Total	10333.95	39				

Table S4. TWO WAY ANOVA analysis for 24h treatment: type of extract vs concentrations in MCF-7 Cells. Sample: (samples 1-4); columns: concentrations (25 -125uM)

ANOVA

Source of Variation	*SS*	*df*	*MS*	*F*	*P-value*	*F crit*
Sample	1365.162	3	455.054	139.4133	1.42E-13	3.098391
Columns	6081.654	4	1520.414	465.8038	1.99E-19	2.866081
Interaction	376.6371	12	31.38643	9.615751	7.24E-06	2.277581
Within	65.28128	20	3.264064			
Total	7888.735	39				

Table S5. TWO WAY ANOVA analysis for 24h treatment: type of extract vs concentrations in MRC-5 Cells. Sample: (samples 1-4); columns: concentrations (25 -125uM)

ANOVA

Source of Variation	*SS*	*df*	*MS*	*F*	*P-value*	*F crit*
Sample	3186.513	3	1062.171	331.603	3.24E-17	3.098391
Columns	2332.277	4	583.0693	182.0305	2E-15	2.866081
Interaction	910.9615	12	75.91346	23.6997	3.31E-09	2.277581
Within	64.06282	20	3.203141			
Total	6493.815	39				

Table S6. TWO WAY ANOVA analysis for 24h treatment: type of extract vs cell type at 25 µM concentration. Sample: (samples 1-4); columns: cell type (HeLa, MCF-7, MRC-5)

ANOVA

Source of Variation	*SS*	*df*	*MS*	*F*	*P-value*	*F crit*
Sample	248.022	3	82.674	13.70089	0.000351	3.490295
Columns	317.4103	2	158.7051	26.30092	4.11E-05	3.885294
Interaction	500.226	6	83.37099	13.8164	9.1E-05	2.99612
Within	72.41045	12	6.034204			
Total	1138.069	23				

Table S7. TWO WAY ANOVA analysis for 24h treatment: type of extract vs cell type at 50 µM concentration. Sample: (samples 1-4); columns: cell type (HeLa, MCF-7, MRC-5)

ANOVA

Source of Variation	*SS*	*df*	*MS*	*F*	*P-value*	*F crit*
Sample	572.0206	3	190.6735	43.79525	9.71E-07	3.490295
Columns	920.6172	2	460.3086	105.7269	2.4E-08	3.885294
Interaction	302.5947	6	50.43245	11.58368	0.000218	2.99612
Within	52.24499	12	4.353749			
Total	1847.477	23				

Table S8. TWO WAY ANOVA analysis for 24h treatment: type of extract vs cell type at 75 µM concentration. Sample: (samples 1-4); columns: cell type (HeLa, MCF-7, MRC-5)

ANOVA

Source of Variation	*SS*	*df*	*MS*	*F*	*P-value*	*F crit*
Sample	1363.461	3	454.4869	129.8195	2.06E-09	3.490295
Columns	1105.473	2	552.7363	157.8834	2.41E-09	3.885294
Interaction	517.8656	6	86.31094	24.65383	4.38E-06	2.99612
Within	42.01096	12	3.500914			
Total	3028.81	23				

Table S9. TWO WAY ANOVA analysis for 24h treatment: type of extract vs cell type at 100 µM concentration. Sample: (samples 1-4); columns: cell type (HeLa, MCF-7, MRC-5)

ANOVA

Source of Variation	*SS*	*df*	*MS*	*F*	*P-value*	*F crit*
Sample	1837.802	3	612.6007	371.5994	4.26E-12	3.490295
Columns	1609.272	2	804.6361	488.0867	3.21E-12	3.885294
Interaction	786.2866	6	131.0478	79.49267	5.86E-09	2.99612
Within	19.78262	12	1.648552			
Total	4253.143	23				

Table S10. TWO WAY ANOVA analysis for 24h treatment: type of extract vs cell type at 125 µM concentration. Sample: (samples 1-4); columns: cell type (HeLa, MCF-7, MRC-5)

ANOVA

Source of Variation	*SS*	*df*	*MS*	*F*	*P-value*	*F crit*
Sample	3031.952	3	1010.651	341.6411	7.01E-12	3.490295
Columns	1989.624	2	994.8119	336.287	2.9E-11	3.885294
Interaction	1443.712	6	240.6186	81.33891	5.13E-09	2.99612
Within	35.49868	12	2.958223			
Total	6500.786	23				

Table S11. TWO WAY ANOVA analysis for 48h treatment: type of extract vs concentrations in HeLa Cells. Sample: (samples 1-4); columns: concentrations (25 -125uM)

ANOVA

Source of Variation	*SS*	*df*	*MS*	*F*	*P-value*	*F crit*
Sample	6652.93	3	2217.643	188.6251	7.83E-15	3.098391
Columns	4076.317	4	1019.079	86.67939	2.43E-12	2.866081
Interaction	1518.243	12	126.5202	10.76137	2.95E-06	2.277581
Within	235.1376	20	11.75688			
Total	12482.63	39				

Table S12. TWO WAY ANOVA analysis for 48h treatment: type of extract vs concentrations in MCF-7 Cells. Sample: (samples 1-4); columns: concentrations (25 -125uM)

ANOVA

Source of Variation	*SS*	*df*	*MS*	*F*	*P-value*	*F crit*
Sample	2046.635	3	682.2117	137.1646	1.66E-13	3.098391
Columns	7537.9	4	1884.475	378.8901	1.53E-18	2.866081
Interaction	244.7131	12	20.39276	4.100141	0.002689	2.277581
Within	99.47343	20	4.973672			
Total	9928.721	39				

Table S13. TWO WAY ANOVA analysis for 48h treatment: type of extract vs concentrations in MRC-5 Cells. Sample: (samples 1-4); columns: concentrations (25 -125uM)

ANOVA

Source of Variation	*SS*	*df*	*MS*	*F*	*P-value*	*F crit*
Sample	3116.045	3	1038.682	276.4	1.92E-16	3.098391
Columns	3175.584	4	793.896	211.2609	4.7E-16	2.866081
Interaction	649.0391	12	54.08659	14.3928	2.65E-07	2.277581
Within	75.15786	20	3.757893			
Total	7015.826	39				

Table S14. TWO WAY ANOVA analysis for 48h treatment: type of extract vs cell type at 25 µM concentration. Sample: (samples 1-4); columns: cell type (HeLa, MCF-7, MRC-5)

ANOVA

Source of Variation	*SS*	*df*	*MS*	*F*	*P-value*	*F crit*
Sample	609.3769	3	203.1256	16.87144	0.000133	3.490295
Columns	1120.411	2	560.2055	46.53019	2.22E-06	3.885294
Interaction	770.9547	6	128.4924	10.67247	0.000324	2.99612
Within	144.4754	12	12.03961			
Total	2645.218	23				

Table S15. TWO WAY ANOVA analysis for 48h treatment: type of extract vs cell type at 50 µM concentration. Sample: (samples 1-4); columns: cell type (HeLa, MCF-7, MRC-5)

ANOVA

Source of Variation	*SS*	*df*	*MS*	*F*	*P-value*	*F crit*
Sample	1176.766	3	392.2554	57.78582	2.1E-07	3.490295
Columns	1848.866	2	924.433	136.1845	5.65E-09	3.885294
Interaction	601.5772	6	100.2629	14.77041	6.49E-05	2.99612
Within	81.4571	12	6.788091			
Total	3708.667	23				

Table S16. TWO WAY ANOVA analysis for 48h treatment: type of extract vs cell type at 75 µM concentration. Sample: (samples 1-4); columns: cell type (HeLa, MCF-7, MRC-5)

ANOVA

Source of Variation	*SS*	*df*	*MS*	*F*	*P-value*	*F crit*
Sample	2076.138	3	692.0459	70.95135	6.62E-08	3.490295
Columns	1071.586	2	535.793	54.93167	9.12E-07	3.885294
Interaction	767.5299	6	127.9217	13.11505	0.000118	2.99612
Within	117.0457	12	9.753808			
Total	4032.299	23				

Table S17. TWO WAY ANOVA analysis for 48h treatment: type of extract vs cell type at 100 µM concentration. Sample: (samples 1-4); columns: cell type (HeLa, MCF-7, MRC-5)

ANOVA

Source of Variation	*SS*	*df*	*MS*	*F*	*P-value*	*F crit*
Sample	2754.973	3	918.3243	535.103	4.88E-13	3.490295
Columns	1581.67	2	790.8349	460.8156	4.51E-12	3.885294
Interaction	941.3597	6	156.8933	91.42094	2.6E-09	2.99612
Within	20.59396	12	1.716164			
Total	5298.597	23				

Table S18. TWO WAY ANOVA analysis for 48h treatment: type of extract vs cell type at 125 µM concentration. Sample: (samples 1-4); columns: cell type (HeLa, MCF-7, MRC-5)

ANOVA

Source of Variation	*SS*	*df*	*MS*	*F*	*P-value*	*F crit*
Sample	3014.384	3	1004.795	261.0037	3.45E-11	3.490295
Columns	2235.901	2	1117.95	290.3968	6.88E-11	3.885294
Interaction	1514.546	6	252.4243	65.56928	1.79E-08	2.99612
Within	46.19681	12	3.849734			
Total	6811.028	23				
